# DYRK1A Kinase Inhibitors Promote β-Cell Survival and Insulin Homeostasis

**DOI:** 10.3390/cells10092263

**Published:** 2021-08-31

**Authors:** Agata Barzowska, Barbara Pucelik, Katarzyna Pustelny, Alex Matsuda, Alicja Martyniak, Jacek Stępniewski, Anna Maksymiuk, Maciej Dawidowski, Ulli Rothweiler, Józef Dulak, Grzegorz Dubin, Anna Czarna

**Affiliations:** 1 Malopolska Center of Biotechnology, Jagiellonian University, Gronostajowa 7A, 30-387 Krakow, Poland; agata.barzowska@doctoral.uj.edu.pl (A.B.); barbara.pucelik@uj.edu.pl (B.P.); katarzyna.pustelny@uj.edu.pl (K.P.); Alex.matsuda@uj.edu.pl (A.M.); grzegorz.dubin@uj.edu.pl (G.D.); 2 Department of Medical Biotechnology, Faculty of Biochemistry, Biophysics and Biotechnology, Jagiellonian University, Gronostajowa 7, 30-387 Krakow, Poland; alicja.martyniak@doctoral.uj.edu.pl (A.M.); jacek.stepniewski@uj.edu.pl (J.S.); jozef.dulak@uj.edu.pl (J.D.); 3 Department of Drug Technology and Pharmaceutical Biotechnology, Medical University of Warsaw, Banacha 1, 02-097 Warszawa, Poland; amaksymiuk1@wum.edu.pl (A.M.); mdawidowski@wum.edu.pl (M.D.); 4 The Norwegian Structural Biology Centre, Department of Chemistry, UiT, The Arctic University of Norway, N-9037 Tromsø, Norway; ulli.rothweiler@uit.no

**Keywords:** DYRK1A, β-cell, kinase, inhibitor, diabetes, hiPSC, organoid, drug development

## Abstract

The rising prevalence of diabetes is threatening global health. It is known not only for the occurrence of severe complications but also for the SARS-Cov-2 pandemic, which shows that it exacerbates susceptibility to infections. Current therapies focus on artificially maintaining insulin homeostasis, and a durable cure has not yet been achieved. We demonstrate that our set of small molecule inhibitors of DYRK1A kinase potently promotes β-cell proliferation, enhances long-term insulin secretion, and balances glucagon level in the organoid model of the human islets. Comparable activity is seen in INS-1E and MIN6 cells, in isolated mice islets, and human iPSC-derived β-cells. Our compounds exert a significantly more pronounced effect compared to harmine, the best-documented molecule enhancing β-cell proliferation. Using a body-like environment of the organoid, we provide a proof-of-concept that small–molecule–induced human β-cell proliferation via DYRK1A inhibition is achievable, which lends a considerable promise for regenerative medicine in T1DM and T2DM treatment.

## 1. Introduction

Stimulation of β-cell regeneration Stimulation of β-cell regeneration with small molecules has emerged as a promising therapeutic strategy against diabetes [1,2,3,4]. Even though endogenous regeneration of β-cells via β-cell replication has the potential to restore cellular mass and actually cure diabetes, the known chemical compounds that promote regeneration or expansion of endogenous β-cells still have inadequate potency for clinical application. Up to date, several β-cell replication-promoting compounds were investigated, including harmine and its analogs [5,6,7,8,9,10], leucettines [11,12], CC-401 [13], 5-iodotubercidin (5-IT) [14], INDY [15] and benzothiazoles derivatives [16], azaindoles and aminopyrazines such as GNF-4877 [17,18,19], VBIT-4, G-1 [3,4] (Figure S1). The common feature of these agents is that they act through inhibition of a dual-specificity tyrosine phosphorylation-regulated kinase 1A (DYRK1A) [20,21,22,23].

Dual-specificity tyrosine-regulated kinases (DYRKs) encompass four subtypes, 1A, 1B, 2, 3 and 4. These CMCG family protein kinases [24] self-activate by auto-phosphorylation of a conserved tyrosine residue and subsequently phosphorylate serine and threonine residues of their respective substrates [24,25,26,27]. DYRK1A is the most extensively studied DYRK kinase. DYRK1A is ubiquitously expressed and participates in biochemical pathways involved in brain development and function, neurodegenerative diseases, tumorigenesis, and apoptosis [24,27,28,29,30]. Additionally, DYRK1A is involved in multiple pathways relevant to pancreatic β-cell proliferation [20,31]. DYRK1A haploinsufficient mice are characterized by severe glucose intolerance and reduced β-cell mass and proliferation, eventually developing diabetes [32,33], but upregulation of DYRK1A in their β-cells only enhances the phenotype [14,34]. As such, DYRK1A has emerged as one of the most attractive therapeutic targets for antidiabetic drugs [14,20,23]. It is now sufficiently documented that inhibition of DYRK1A in pancreatic β-cells leads to enhanced proliferation and promotes glucose-stimulated insulin secretion (GSIS) without the risk of hypoglycemia. However, the small molecule toolset available and understanding of DYRK1A inhibition in diabetes is still insufficient.

Harmine was the first identified small molecule inhibitor of DYRK1A and remains the gold standard in β-cell proliferation. Simultaneous inhibition of DYRK1A and SMADs with harmine leads to a remarkable rate of human β-cell proliferation both in vivo and in vitro [35]. The combination of harmine and TGF-β signaling inhibitors has demonstrated a synergistic effect on β-cells proliferation and function [36,37]. The average replication rates after such treatment rise by 5% to 8%. It is an effect that has never been observed with any other β-cell stimulants. This by far exceeds the rates observed in physiological pancreatic β-cell replication in the first year of life [38]. Over the last decade, many DYRK1A inhibitors were reported, but none has reached the clinical trials yet [5,8,10,18,21,22,39,40,41,42,43].

**Figure 1:**
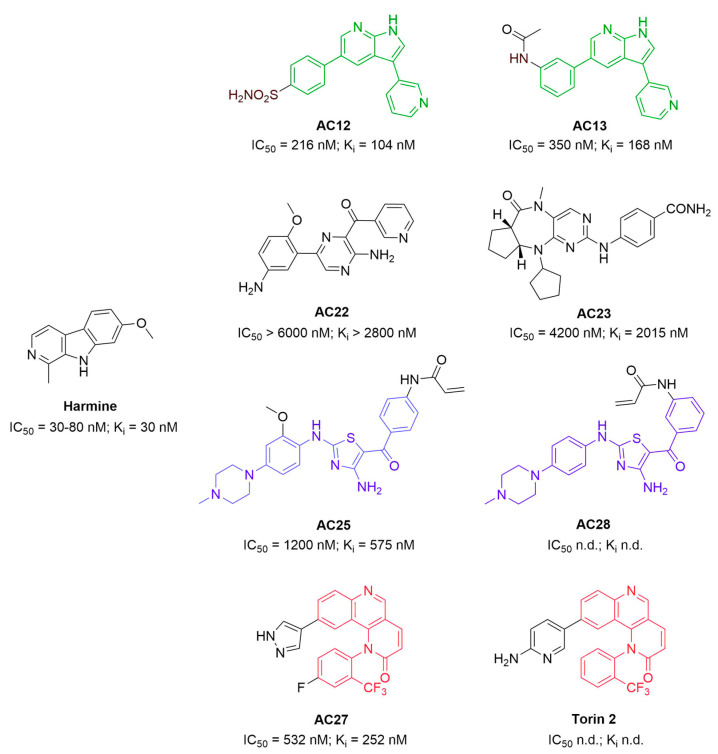
Chemical structures of the investigated DYRK1A inhibitors. IC50 and Ki values are referenced in Czarna et al. [44].

In search for inhibitors of tau phosphorylation, a neuronal pathology dependent on DYRK1A kinase and a hallmark of Alzheimer’s Disease, we have previously identified several DYRK1A inhibitors of diverse scaffolds [44] (examples shown in Figure 1). The inhibitors showed DYRK1A inhibition (Ki values) between 200 nM 2800 nM [44], and kinase profiling demonstrated that some are highly specific against DYRK1A and CLK2 kinases. Functionally, the inhibitors prevented tau phosphorylation demonstrating an on-target effect in vitro [44]. 

Here, we report DYRK1A inhibitors evaluation at the cellular, ex vivo, and human organoid level. The compounds potently promote human β-cell proliferation and enhance insulin secretion. Characterization of the involved pathways supports the on-target effects of tested compounds. The effects are more pronounced than the current gold standard harmine, providing significant improvement to the available toolset of small molecules with β-cell regenerating potential.

## 2. Materials and Methods

### 2.1. Materials

All DYRK1A inhibitors: 

4-(3-(pyridin-3-yl)-1H-pyrrolo[2,3-b]pyridin-5-yl)benzenesulfonamide (AC12); 4-(3-pyridin-3-yl-1H)-pyrrolo[2,3-b]pyridin-5-yl)acetanilide (AC13); (3-azanyl-6-(5-azanyl-2-methoxy-phenyl)pyrazin-2-yl)-pyridin-3-yl-methanone (AC22); 4-(((6aS,9aR)-10-cyclopentyl-5-methyl-6-oxo-5,6,6a,7,8,9,9a,10-octahydrocyclopenta[e]pyrimido[5,4-b][1,4]diazepin-2-yl)amino)benzamide (AC23); *N*-(4-((4-azanyl-2-((2-methoxy-4-(4-methylpiperazin-1-yl)phenyl)amino)-1,3-thiazol-5-yl)carbonyl)phenyl)propenamide (AC25); 1-(4-fluoro-2-(trifluoromethyl)phenyl)-9-(1H-pyrazol-4-yl)benzo[h][1,6]naphthyridin-2-one (AC27); *N*-(3-((4-azanyl-2-(4-(4-methylpiperazin-1-yl)phenyl)amino)-1,3-thiazol-5-yl)carbonyl)phenyl)propenamide (AC28); were kindly provided by Prof. Nathanael Grey (Dana-Farber Cancer Institute, Boston, MA, USA). Torin2 and harmine were purchased from Sigma−Aldrich (Burlington, MA, United States). Fetal Bovine Serum (FBS) and antibiotics for cell culture, culture growth media and reagents were from PAN-BioTech GmbH (Aidenbach, Germany). Ethanol (EtOH), methanol (MeOH), DMSO and Triton X-100 were supplied from Avantor Performance Materials Poland SA (Gliwice, Poland).

### 2.2. Protein Expression and Purification

The fragment of the gene encoding the kinase domain of DYRK1A (126–490) was cloned into pET24a with C-terminal His-tag. Protein expression was carried out in *E. coli* BL21 (DE3) RIL (Stratagene) strain in LB medium with kanamycin (50 μg/mL) at 17 °C for 16 h. The pellet was resuspended in cold lysis buffer (20 mM HEPES pH 7.5 with 500 mM NaCl, 5% glycerol, 15 mM imidazole and 5 mM β-Mercaptoethanol supplemented with EDTA-free Protease Inhibitor Cocktail (Roche)) and the cells were disintegrated by sonication. The clear cell lysate was passed through HisPur™ Cobalt resin (ThermoFisher Scientific, Waltham, MA, United States), and bound protein was eluted stepwise with imidazole (50–300 mM). The fraction corresponding to DYRK1A was pulled and dialyzed against the final buffer (20 mM HEPES pH 7.5, 50 mM NaCl and 5 mM β-Mercaptoethanol). Final purification was carried out via ion-exchange chromatography on HiTrap Q FF column (Cytiva Life Sciences, Marlborough, MA, United States) followed by size exclusion chromatography on HiLoad 16/600 Superdex 75 pg column (Cytiva Life Sciences, Marlborough, MA, United States). The purified DYRK1A kinase was flash-frozen in liquid nitrogen and kept at −80 °C for further analysis.

### 2.3. MST Measurements

To determine compound-DYRK1A kinase binding affinity, purified His-tagged kinase domain was labeled using RED-Tris-NTA 2nd Generation Labeling Kit (NanoTemper, Munich, Germany). Dissociation constants were determined using a direct binding assay in which 10 µL of the unlabeled compound in a concentration ranging from 250 µM to 8 nM was incubated with 10 µL of 20 nM of labeled DYRK1A. All measurements were performed in triplicates and carried out using Monolith NT. 115 instrument (NanoTemper, Munich, Germany) in PBS-T buffer (137 nM NaCl, 2.5 mM KCl, 10 mM Na_2_HPO_4_, 2 mM KH_2_PO_4_ pH 7.4 with 0.005% Tween-20) at room temperature with 80% excitation and 40% MST power. The Kd values were calculated using MO Affinity Analysis software (NanoTemper, Munich, Germany).

### 2.4. Cook Assay

The inhibitory potency (Ki) of all compounds was determined in the ATP regenerative NADH consuming DYRK1A activity assay. The Cook assay was carried out as described previously [45] with ATP and DYRKtide peptide (RRRFRPASPLRGPPK) used as a substrate. Briefly, the 75 µL of assay mixture (100 mM MOPS pH 6.8, 100 mM KCl, 10 mM MgCl_2_, 1 mM phosphoenolpyruvate, 1 mM DYRKtide, 1mM β-Mercaptoethanol, 15 U/mL lactate dehydrogenase with 10 U/mL pyruvate kinase and 10.7 mM NADH) was mixed with 10 µL of 5 µM DYRK1A kinase and 5 µL of the compound in DMSO in a concentration ranging from 200 µM to 20 nM and incubated for 10 min at room temperature. Then 10 µL of 1280 µM ATP was added to start the reaction. The enzyme velocity was measured at 340 nm over a time period of 300 s at room temperature. All measurements were carried out in triplicate, and IC_50_ was determined using GraphPad Prism software, and Ki values were calculated using the Cheng-Prusoff equation with a KM value for DYRK1A of 118 µM.

### 2.5. NFAT Luciferase Reporter Assay

The Renilla luciferase reporter assay was performed in HEK 293 NFAT cell line, constitutively expressing DYRK1A, NFAT and Renilla luciferase under the control of an NFAT-RE promoter [44]. The cells were seeded at 10,000 per well in 96-well white plates (Cellstar, Greiner; Burlington, MA, United States). The following day cells were incubated with the indicated concentration of inhibitor for 30 min, then the medium was changed, and cells were stimulated with 1 μM ionomycin and 10 nM PMA for 5 h. Next, the cell lysate was harvested to measure luciferase activity with Renilla-Glo Luciferase Assay System (Promega, Wisconsin, United States). All experiments were carried out in triplicates.

### 2.6. Insulinomas Cell Culture

Rat insulinoma (INS-1E) cells were kindly provided by Prof. P. Maechler [46]. INS-1E were grown in DMEM (Pan Biotech) with the addition of 10% FBS (Pan Biotech) and supplemented by antibiotics (100 IU mL^−1^ penicillin and 100 mg· mL^−1^ streptomycin) and 55 µM β-Mercaptoethanol (Pan Biotech, Aidenbach, Germany). Mouse insulinoma (MIN6) cells were grown in DMEM (Pan Biotech) with the addition of 15% FBS (Pan Biotech) and supplemented by antibiotics (100 IU mL^−1^ penicillin and 100 mg·mL^−1^ streptomycin) and 55 µM β-Mercaptoethanol (Pan Biotech). The cells were cultured in incubators maintained at 37 °C with 5% CO_2_ under fully humidified conditions. All experiments were performed on cells in the logarithmic phase of growth. Media were replaced every 2 days, and cells were subcultured using 0.25% trypsin-EDTA (Gibco).

### 2.7. Cytotoxicity and Cell Survival Assay

The MTT (3-(4,5-dimethylthiazol-2-yl)-2,5-diphenyl tetrazolium bromide) (Invitrogen) assay was used to quantify cell survival and inhibitor-mediated cytotoxicity. After cell attachment, compounds in the growth medium at concentrations from 0 to 100 μM were added to the cell cultures. In other experiments 5 μM solution of DYRK1A inhibitor and 1, 5, or 10 μM solution of TGF-β inhibitor was added to the cell cultures. The treated cultures were incubated for 24 h. Next, the compounds’ solutions were removed, cells were washed in PBS, and a fresh culture medium with FBS and antibiotics was added to each well. Next, MTT dissolved in PBS (Pan Biotech) at content 10% of final solution were added to each well, and the microplates were further incubated for 3 h. The medium was then discarded, and 100 μL of a mixture of DMSO/methanol (1:1) was added to the cultures and mixed thoroughly to dissolve the dark blue crystals of formazan. Formazan quantification was performed using an Infinite M200 Reader (Tecan) automatic microplate reader by absorbance measurements with a 565 nm test wavelength.

### 2.8. Glucose-Stimulated Insulin Secretion (GSIS)

Cells were cultured in a low glucose medium (5.5 mM) for 24 h. After this medium was changed to lower glucose medium (2 mM) for 2 h and then for high glucose medium (25 mM) for 30 min, media were harvested and/or cells were used for further analysis.

### 2.9. Staining for Insulin, Ki67, Glucagon, C-peptide and DYRK1A

The presence of insulin, glucagon, C-peptide, Ki67, and DYRK1A was assessed in MIN6 and INS-1E pancreatic cell lines. Before imaging, cells were seeded onto slides at a density of 1 × 10^5^ cells and maintained at 37 °C in 95% atmospheric air and 5% CO_2_ in a humidified atmosphere for 24 h. After washing with fresh medium, cells were incubated with 5 µM inhibitor solution for 24 h. After this time, the solution was replaced with a culture medium for another 48 h. The cells were fixed. Then after washing and permeabilization, they were incubated with specific antibodies diluted in PBS. Next, the cells were incubated for 10 min with Hoechst33342 (Sigma-Aldrich). After incubation, at 37 °C, in the dark, the cells were washed twice with HBSS (Gibco), the slide was transferred to a microscope table, and the cells were visualized under a Zeiss LSM 880 confocal microscope (Carl Zeiss, Jena, Germany) with a 63× immersion objective. Images were analyzed by Zeiss ZEN Software.

### 2.10. Flow Cytometry Analysis

The presence of insulin, glucagon, C-peptide, Ki67 and DYRK1A was assessed in MIN6 and INS-1E pancreatic cell lines. Before analysis, cells were seeded onto slides at a density of 1 × 105 cells and maintained at 37 °C in 95% atmospheric air and 5% CO_2_ in a humidified atmosphere for 24 h. After washing with fresh medium, cells were incubated with 5 µM inhibitor solution for 24 h. After this time, the solution was replaced with a culture medium for another 48 h. The cells were then detached from the plate using trypsin. The cells were fixed. Then after washing and permeabilization, they were incubated with specific antibodies diluted in PBS. Cells were analyzed with BDAccuri flow cytometer and calculated using FlowJo software (Ashland, Oregon-based FlowJo LLC, a subsidiary of Becton Dickinson).

### 2.11. Cell Cycle Analysis

To synchronize cell cultures, we used the serum starvation protocol. INS-1E cells were seeded in a 6-well plate in a growth medium with 20% FBS overnight. Then the cultures were rinsed by PBS and change to a serum-free medium. After serum starvation for 24 h, cells were released into the cell cycle by the addition of serum and 5 µM inhibitor solution for 24 h. The cells were then detached from the plate using trypsin. Then after washing, they were incubated with 50 µg/mL propidium iodide for 15 min in the dark. Cells were analyzed with the BDAccuri flow cytometer and calculated using FlowJo software.

### 2.12. Differentiation hiPSC into Pancreatic Islets Cells

Differentiation of hiPSC into iPSC-derived β-cell islets was carried out following the protocol established by Pellegrini et al. for pluripotent stem cells with slight modifications. A day before differentiation, 800,000 cells were seeded on coated with Geltrex (Gibco) 12 wells plate in Essential 8 medium (Gibco) with Y-27632 10 µM (Abcam). The following culture media were used for differentiation:

M1 medium: MCDB131 (Gibco) + 8 mM d-(þ)-Glucose (Sigma) + 1.23 g/L NaHCO_3_ (Sigma) + 2% BSA (Sigma) + 0.25 mM Vitamin C (Sigma) + 1% Pen/Strep (Lonza, Basel, Switzerland) + 1% l-glutamine (Lonza);

M2 medium: MCDB131 + 20 mM d-Glucose + 1.754 g/L NaHCO_3_ + 2% BSA + 0.25 mM Vitamin C + Heparin 10mg/mL (Sigma) + 1% Pen/Strep + 1% l-glutamine. All media were filter-sterilized through a 0.22 µm bottle top filter (Corning, New York, NY, USA). For sequential culture medium changes, small molecules and growth factors were added to the base media immediately before daily exchange. Media switches were as follows: days 0–3: STEMdiff™ Definitive Endoderm Kit (STEMCELL, Vancouver, Canada) used following manufacturer instructions; days 4–6: M1 medium + 50 ng/mL KGF (Peprotech, London, UK) + 1:50,000 ITS-X (Invitrogen, Carlsbad, CA, USA); days 7–8: M1 medium + 50 ng/mL KGF + 0.25 µM Sant1 (Sigma) + 2 mM Retinoic acid (RA) (Sigma) + 500 nM PdBU (Millipore, Berlington, MA, United States) + 1:200 ITS-X + 200 nM LDN193189 (only Day 7) (Sigma); days 9–13: M1 medium + 50 ng/mL KGF + 0.25 µM Sant1 + 100 nM RA + 1:200 ITS-X; days 14–18: M2 medium + 0.25 µM Sant1 + 100 nM RA + 1 µM XXI (Millipore) + 10 µM Alk5i II (Selleckchem, Munich, Germany) + 1 µM l-3,30,5-Triiodothyronine (T3) (Sigma) + 20 ng/mL Betacellulin (R&D, Minneapolis, MN, USA) + 1:200 ITS-X.

### 2.13. IPSC Staining Protocol—3D Cell Culture—Organoids

The presence of insulin and glucagon was assessed in organoids (3D cell culture). Organoids were fixed in PFA (3.8%) for 30 min, washed with PBS and 0.1% Triton X-100 was added for 1.5 h. Then, cells were washed with PBS, and samples were transferred to 1% BSA + 0.05% Triton X-100 solution for 3 h. After this time, organoids were incubated with primary antibodies for 12 h at 4 °C. The secondary antibody was then added for 3 h. Next, the samples were incubated for 10 min with Hoechst33342. After incubation, at 37 °C, in the dark, the organoids were washed twice with HBSS, the slide was transferred to a microscope table and the cells were visualized under a Zeiss LSM 880 confocal microscope (Carl Zeiss, Jena, Germany) with a 63× immersion objective. Images were analyzed by Zeiss ZEN Software (Carl Zeiss, Jena, Germany).

### 2.14. ELISA Assay

The Insulin ELISA (Ultra Sensitive) was used to quantify insulin levels in control and treated organoids and isolated islets from the mouse pancreas (Crystal Chem, Downers Grove, IL, USA). Islets were homogenized in lysis buffer. Obtained buffer lysates and medium samples (after GSIS) were analyzed for total protein level and subsequently processed following the manufacturer’s protocol.

### 2.15. Mouse Islet Isolation

Mice (male, initially 12 weeks old) were sacrificed by cervical vertebral dislocation. Before starting the incision, their bodies were sprayed with 70% EtOH. A hemostatic clamp was placed on both sides of the small intestine, leaving a small pocket to absorb the collagenase solution into the intestine. Next, 3 mL of cold collagenase solution was introduced into the pancreas through the bile duct. The pancreas was removed from the body and placed in a falcon tube containing 2 mL of collagenase solution. The tube was placed in a water bath at 37 °C. It was incubated for 13 min. At the end of this time, the falcon was shaken vigorously. The collagenase was inactivated with FBS. The sample was centrifuged for 30 s (300× *g*). This step was repeated 3 times. HBSS was then added to the sample and centrifuged two more times at the same speed as before. After the last centrifugation, HBSS was removed and Percoll (1.045 g/mL) (Sigma-Aldrich) was added. The Percoll solution was left to sediment for 5 min. After this time, the islets were collected from the bottom of the falcon. The step was repeated 3 times. Finally, islets were cultured in RPMI-1640 (Pan Biotech) supplemented with 2 mM l-glutamine (Gibco), 10% fetal bovine serum (FBS) and 1% penicillin/streptomycin and the culture medium was changed daily.

### 2.16. Mouse Islet Staining Protocol

The presence of insulin, C-peptide, glucagon, Pdx-1 and Nkx6.1 was assessed in isolated mouse islets. Islets were fixed in PFA (3.8%) for 30 min, washed with PBS and 0.15% Triton X-100 was added for 1.5 h, then washed again with PBS and samples were transferred to 1% BSA + 0.05% Triton X-100 solution for 3 h. After this time, islets were incubated with primary antibody for 12 h at 4 °C. The secondary antibody was then added for 3 h. Next, the islets were incubated for 10 min with Hoechst33342. After incubation, at 37 °C, in the dark, the samples were washed twice with HBSS, the slide was transferred to a microscope table and the cells were visualized under a Zeiss LSM 880 confocal microscope (Carl Zeiss, Jena, Germany) with a 63× immersion objective. Images were analyzed by Zeiss ZEN Software.

### 2.17. Statistical Analysis

Data are presented as mean ± standard deviation (SD) or standard error of the mean (SEM of N independent experiments (at least N = 3). Statistical significance of differences was assessed using GraphPad Prism 5.0 (GraphPad Software, San Diego, CA, USA). The differences between groups were compared using a two-way ANOVA, and *p* < 0.05 was considered significant.

## 3. Results

### 3.1. Binding Affinity and Inhibitory Potential of Selected DYRK1A Inhibitors

DYRK1A inhibitors representing diverse chemical scaffolds [44] (Figure 1) were selected to analyze the possible influence of DYRK1A inhibition on β-cell proliferation. Harmine and Torin2 were used for reference. The latter compound, a known mTOR inhibitor, was included because of a high degree of structural similarity to one of our lead inhibitors, AC27.

**Figure 2:**
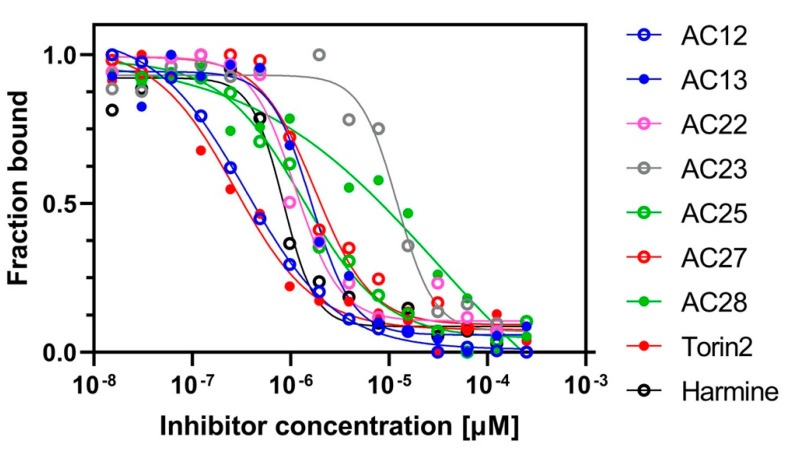
Direct binding determined by MST. Changes in normalized fluorescence signal upon temperature jump at different inhibitor concentrations were converted to inhibitor fraction bound and plotted against concentration. The average of five measurements for each inhibitor concentration is shown. Standard deviation bars are omitted for clarity. The Hill equation was fit to the experimental data.

Microscale thermophoresis (MST) measures the direct interaction of tested compounds and DYRK1A (Table 1, Figure 2). We determined the dissociation constants in the direct binding assay using a constant concentration of fluorescent-labelled DYRK1A kinase domain (20 nM) titrated with increasing concentrations of compounds (15 nM-250 µM). AC12, AC13, harmine and Torin2 showed sub-micromolar K_d_ values (<1 µM). AC25, AC27 and AC22 were characterized by K_d_ values in the low micromolar range (1 µM to 2.5 µM), while the K_d_ values for AC23 and AC28 exceeded 10 µM.

We evaluated the inhibition of DYRK1 mediated phosphorylation using the Cook activity assay [45]. We checked the potency (Ki) of each tested compound using the fixed concentration of DYRK1A (0.5 µM), ATP (128 µM) substrate peptide of DYRK1A—DYRKtide (RRRFRPASPLRGPPK, 1 mM), and increasing concentration of compound (20 Nm–200 µM) [44]. All tested small molecules inhibited the activity of DYRK1A in an ATP-competitive manner with the Ki values below 5 µM. The most active compounds (AC12, AC13, harmine and AC27) inhibited the phosphorylation of the substrate peptide with the Ki values below 200 nM, while the Ki values of the remaining compounds were in the range of 1 µM to 5 µM (Table 1, Table S1).

The correlation between K_d_ values, characterizing the interaction of tested compounds with DYRK1A as determined by MST, and Ki, reflecting the inhibitory potential in Cook assay, was satisfied with only Torin2 being an outlier (Figure S2). This demonstrates that both methods are suitable for compound ranking. Nevertheless, the determined K_d_ values differed by roughly an order of magnitude for the determined Ki demonstrating the limitations of the methods most likely related to the labelling necessary in the MST assay.

### 3.2. NFAT Luc Reporter Assay

**Figure 3:**
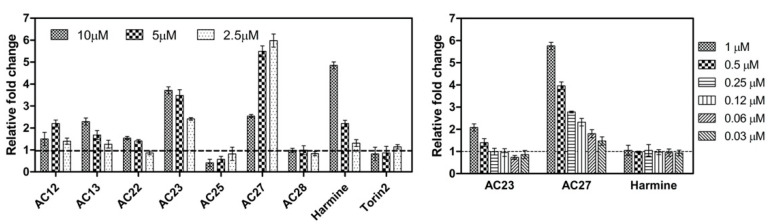
Effect on NFAT activity (luciferase reporter assay). Results are normalized at the basal activity of the luciferase (mock treated cells; CTRL). Application of 1 µM of AC27 leads to the same activity as 10 µM of harmine, making the former nearly 10 times more active in this particular cellular assay. Data are expressed as mean ± SD from 3 independent experiments.

The DYRK1A activity and pancreatic β-cell proliferation are linked via the NFAT (Nuclear factor of activated T-cells) transcription factor that plays a major role in regulating the cell cycle [47]. DYRK1A phosphorylates NFAT, restraining its nuclear translocation and activity regardless of the intracellular Ca^2+^ influx and calcineurin activation [48]. Therefore, a coupling between inhibition of DYRK1A and activation of NFAT is expected. We measured the impact of AC compound series on NFAT activity in cell-based luciferase reporter assay [44]. A HEK293 cell line expressing DYRK1A was incubated with various concentrations of inhibitors, and the activity of NFAT-regulated luciferase reporter was assessed (Figure 3). Torin2, AC28 and AC25 did not promote NFAT-activity. In fact, the activity dropped below the basal level in the presence of the above compounds, which may be attributed to toxicity (Figure S3). On the other hand, compounds AC13, AC22, AC23 and harmine induced NFAT-controlled luciferase activity in a dose-dependent manner. AC27 was the most active compound in the low concentration range (0.03 µM–1 µM; below significant toxicity level), inducing up to a 6-fold increase of NFAT activity, while the effect of harmine has not even reached significance at the comparable concentration range.

### 3.3. Inhibition of DYRK1A Activity Stimulates Growth of INS-1E and MIN6 β-Cells

Prior to further evaluations, we investigated whether harmine, Torin2 and the DYRK1A inhibitors influence the INS-1E and MIN6 cell viability. In this experiment, we also included Torin2, a structural analog of AC27. Torin2 is a strong and selective ATP-competitive inhibitor of mTOR, a key regulator of cell growth, survival and autophagy. The compound is extensively studied in cancer research because it effectively inhibits the proliferation of lung, breast, colorectal and cervical cancer cell lines. In addition, the compound blocks the mTOR complex 1 (mTORC1)-associated cell cycle progression and induces autophagy in hepatocellular carcinoma cells [49,50]. We expected that the mentioned activities may influence the viability of INS-1E and MIN6 cell lines, thus rendering the results of subsequent experiments difficult to interpret.

**Figure 4:**
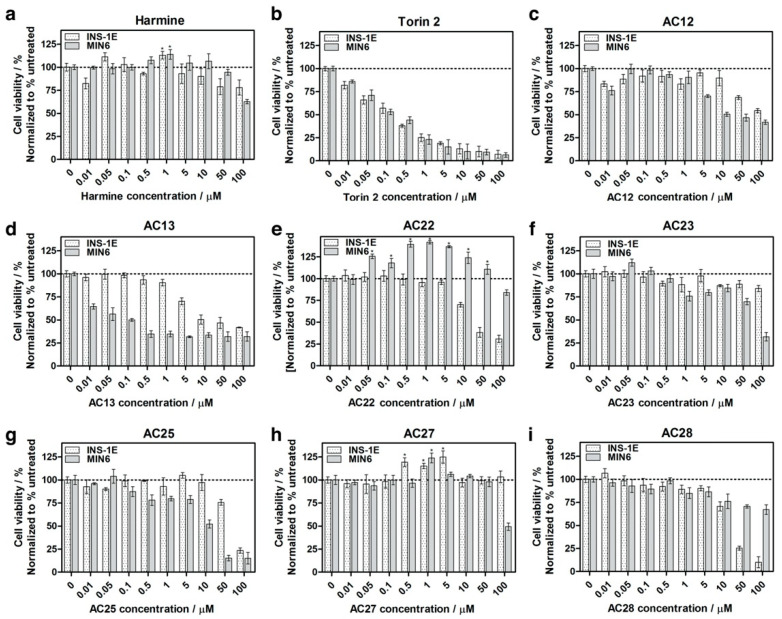
Relative viability of the MIN6 and INS-1E cell lines treated with Torin2, harmine and DYRK1A inhibitors in an MTT assay. The concentrations of the compounds ranged from 0 μM to 100 μM for (**a**) harmine, (**b**) Torin2, (**c**) AC12, (**d**) AC13, (**e**) AC22, (**f**) AC23, (**g**) AC25, (**h**) AC27 and (**i**) AC28. Data are expressed as mean ± SEM, * *p* < 0.05 as compared to the control.

In our experiment, harmine did not show adverse effects on the viability of INS-1E cells even at high concentrations (Figure 4a). However, as expected, the mTOR inhibitor Torin2 was toxic towards β-cell lines and significantly reduced INS-1E viability when applied in concentrations higher than 0.05 µM (Figure 4b). The compound was therefore excluded from the subsequent experiments. In contrast to the marked decrease in cell viability caused by Torin2, other tested compounds did not show significant toxic effects at concentrations below 10 µM (Figure 4c–f).

**Figure 5:**
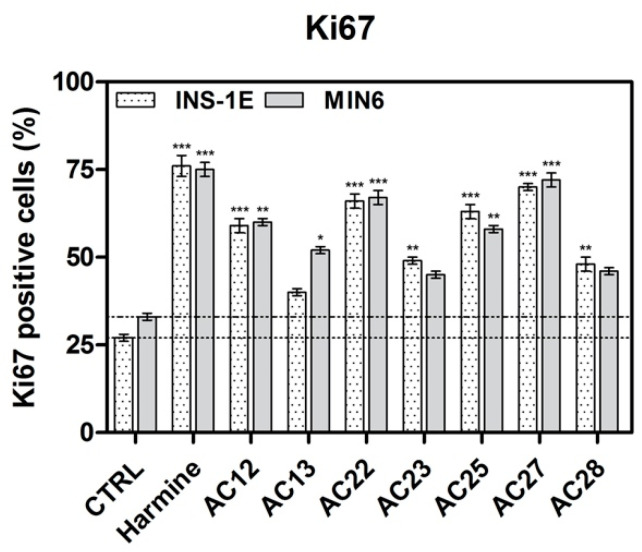
Analysis of the influence of DYRK1A inhibitors on proliferation of INS-1E and MIN6 cells. In Ki67 staining, 5 µM of each inhibitor and harmine as a positive control were used. Data are expressed as mean ± SEM; * *p* < 0.05, ** *p* < 0.01, *** *p* < 0.001 compared to the control.

To study the impact of DYRK1A on β-cell replication, we incubated INS-1E and MIN6 cells with DYRK1A inhibitors (Figure 5). The rate of proliferation of INS-1E and MIN6 cells was assessed by the Ki67 staining analysis. The level of cell proliferation is closely related to the expression of the Ki67 protein. Under normal conditions, the protein is present in the active phase of the cell cycle, except in G0 and early G1 [51]. Therefore, it is an excellent marker of the growth fraction of the cell population. All tested DYRK1A inhibitors promoted survival and replication of the INS-1E cells, implying that they may support the net growth of INS-1E and MIN6 cells. In fact, the cell cultures treated with inhibitors for 3–12 days grew faster than the untreated cell cultures since the former cultures contained more cells (Figure S4).

### 3.4. DYRK1A Inhibition Mediated by the Investigated Compounds Increases β-Cell Proliferation

**Figure 6:**
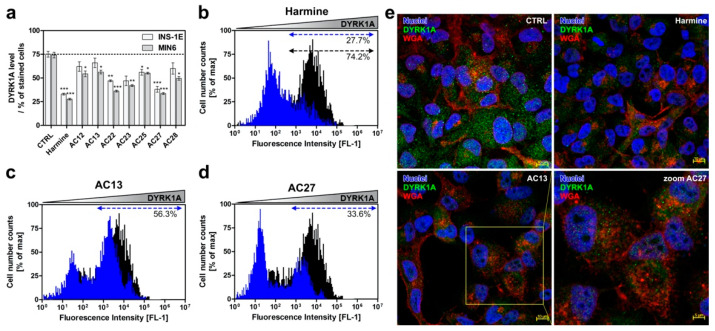
DYRK1A expression in the investigated rodent cell lines. (**a**–**d**) Flow cytometry analysis of DYRK1A expression in MIN6 cell line. After incubation of cells with inhibitors (24 h), the cells were stained with a DYRK1A antibody. (**a**) DYRK1A expression levels in cells treated with the investigated compounds; data are expressed as % of max in control cells. (**b**) DYRK1A expression levels in control cells (black) and harmine-treated cells (blue). (**c**) DYRK1A expression levels in control (black) and AC13-treated cells (blue). (**d**) DYRK1A expression levels in control (black) and AC27-treated cells (blue). (**e**) Immunofluorescence double-staining with antibodies against DYRK1A (green) and cell membrane (red) of MIN6 cell line without (CTRL) and after treatment with harmine (upper right) and AC27 (lower panel). Cell nuclei were stained with Hoechst 33,342 (blue). Scale bars, 10 μm and 5 μm for zoom, respectively. Data are expressed as mean ± SEM, N = 3; * *p* < 0.05, ** *p* < 0.01; *** *p* < 0.001 as compared to control.

We hypothesized that treatment of β-cells with DYRK1A inhibitors would augment their regeneration and restore their functions. We used INS-1E rat insulinoma cells and MIN6 cells derived from a mouse insulinoma that display the β-cell characteristics, including insulin secretion in response to, e.g., glucose. They share many features with the primary β-cells and are, therefore, widely used as model cells to study their function [52]. We performed immunofluorescence analysis to measure the DYRK1A expression in INS-1E and MIN6 cell lines (Figure 6). As expected, DYRK1A is highly expressed in both cell lines (Figure 6a). In addition, we observed that the inhibitors affected the fluorescence signal observed in DYRK1A positive cells. The flow cytometry analysis (Figure 6b–d) indicated a decreased fluorescence after treatment with DYRK1A inhibitors (for instance, 74% vs. 27.7% after treatment with harmine, 56.3% and 33% for AC13 and AC27, respectively). Accordingly, we obtained similar results in the confocal imaging experiment, in which the highest DYRK1A signal appeared in control cells (untreated) and was reduced after treatment with harmine (positive control) or AC27 (Figure 6e).

### 3.5. The Influence of DYRK1A Inhibitors on the INS-1E and MIN6 Functionality

**Figure 7:**
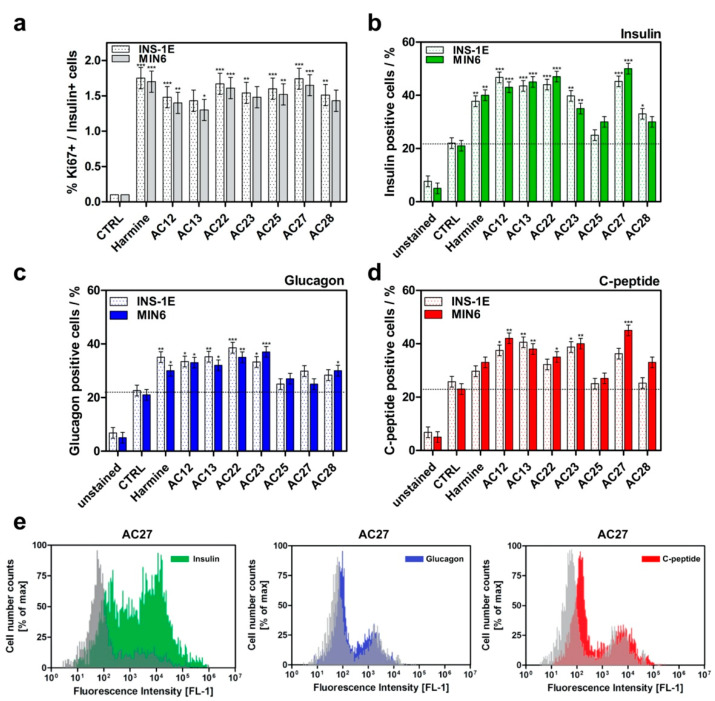
The influence of DYRK1A inhibitors on INS-1E and MIN6 function: Flow cytometry analysis of insulin, C-peptide and glucagon expression in MIN6 and INS-1E cell lines. 24 h after incubation with 5 μM of the inhibitors and 72 h after applying the compounds, the cells were stained with the appropriate antibodies. (**a**). Effects of inhibitors on Ki67 immunolabeling in MIN6 and INS-1E cell lines. (**b**) Effects of inhibitors on insulin immunostaining in MIN6 and INS-1E cell lines. (**c**) Effects of inhibitors on C-peptide immunostaining in MIN6 and INS-1E cell lines. (**d**) Effects of inhibitors on glucagon immunostaining in MIN6 and INS-1E cell lines. (**e**) Insulin, Glucagon and C-peptide level in INS-1E cells treated with AC27 compared with control (grey). Data are expressed as mean ± SEM. * *p* < 0.05, ** *p* < 0.01; *** *p* < 0.001 as compared to the control.

DYRK1A inhibition has been reported to drive human pancreatic β-cell proliferation. Therefore, we investigated the effect of DYRK1A inhibitors on insulin-positive β-cell proliferation (Figure 7a). The tested inhibitors induced β-cell proliferation at 5 μM compared to the untreated cells, as quantified by increased Ki67 labeling of insulin-labeled cells. The data show that AC22 and AC27 were most potent in inducing β-cell proliferation and produced an effect similar to that produced by harmine used as a positive control.

**Figure 8:**
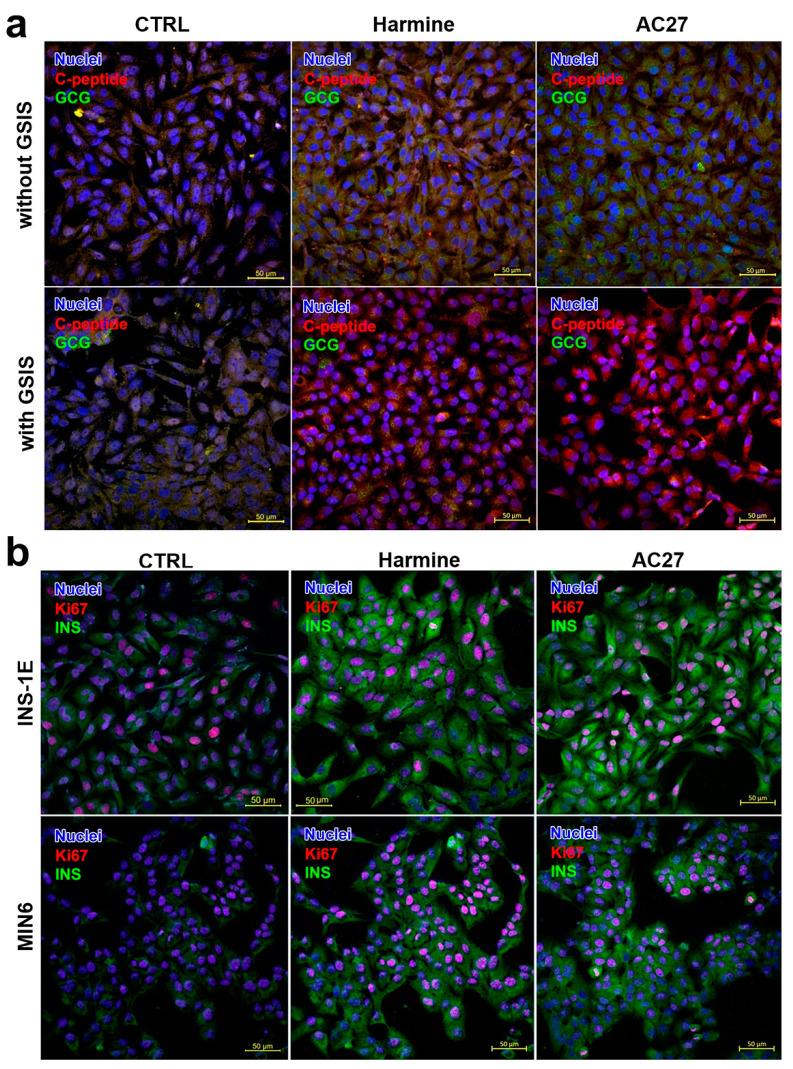
Representative confocal images. (**a**) Proliferation rate (Ki67) and insulin (INS) secretion in INS-1E and MIN6 cells for cells not treated with inhibitor (CTRL), harmine and AC27. (**b**) Expression levels of C-peptide and glucagon (GCG) before and after GSIS. Scale bars, 50 μm.

Next, we tested DYRK1A inhibitors in an in vitro GSIS assay (Figure 7). Harmine was used as a control as it causes enhanced insulin secretion and increases C-peptide levels (Figure S5). Treatment of INS-1E and MIN6 cells with each compound increased insulin release at low- and high-glucose challenges. The compounds caused a 15–50% increase in insulin release. Noteworthy, in the case of AC27 treatment, we observed a balanced (lower) glucagon level (Figure 7c). Moreover, the compound produced more appropriate pancreatic hormone levels (higher insulin/C-peptide secretion and lower glucagon level after GSIS protocol as compared to harmine), as observed by flow cytometry (Figure 7e) and confocal microscopy (Figure 8).

### 3.6. The Combination of AC27 with TGF-β Inhibitors Improves the Functionality of β-Cells In Vitro

**Figure 9:**
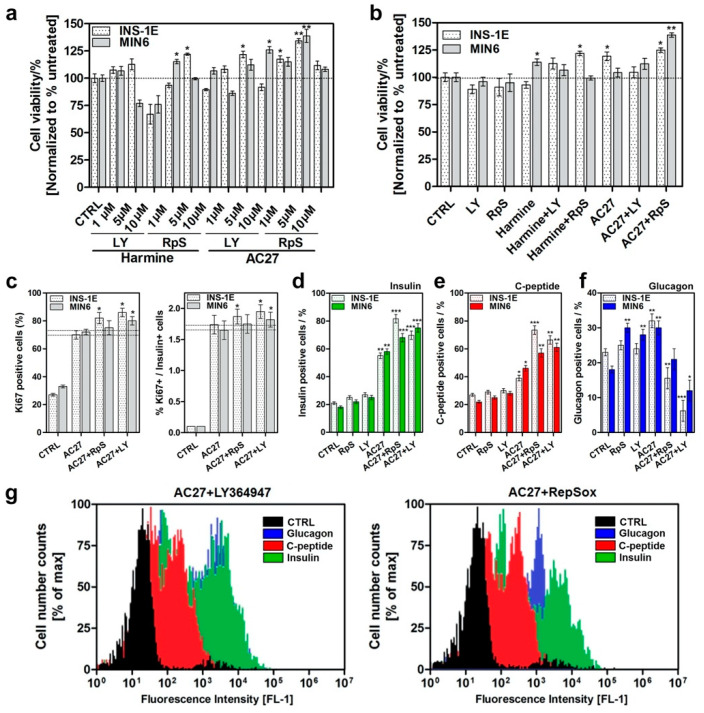
Effects of the AC27-TGF-β combination on INS-1E and MIN6 cells. (**a**) Viability of MIN6 and INS-1E cell lines after treatment with the harmine-TGF-β inhibitor and AC27-TGF-β inhibitor combination. The concentration of TGF-β inhibitors used was 1 μM, 5 μM or 10 μM. The concentration of DYRK1A inhibitors used was 5 μM. Viability (after 24 h) of MIN6 and INS-1E cell lines after treatment with a combination of harmine-TGF-β inhibitor and AC27-TGF-β inhibitor and with the use of DYRK1A or TGF-β inhibitors alone. (**b**) Analysis of the influence of the AC27-TGF-β inhibitor combination on the β-cell proliferation: INS-1E and MIN6 proliferation assay using 5 μM of each compound (DYRK1A and TGF-β inhibitor). (**c**) Effects of the AC27-TGF-β inhibitor combination on Ki67 immunolabeling in MIN6 and INS-1E cell lines. (**d**) Effects of the AC27-TGF-β inhibitor combination on insulin immunostaining in MIN6 and INS-1E cell lines. (**e**) Effects of the AC27-TGF-β inhibitor combination on the C-peptide immunostaining in MIN6 and INS-1E cell lines. (**f**) Effects of the AC27-TGF-β inhibitor combination on glucagon immunostaining in MIN6 and INS-1E cell lines. (**g**) Representative flow cytometry profiles from analysis summarized in panels (**d**–**f**) in INS-1E cells. Data are expressed as mean ± SEM. * *p* < 0.05, ** *p* < 0.01; *** *p* < 0.001 as compared to the control.

In 2019, Wang, et al. [35] demonstrated that the combined pharmacologic or genetic inhibition of DYRK1A and TGF-β signaling significantly improves β-cell proliferation in vitro and in vivo [35]. Thus, we explored the effects of two most common and non-cytotoxic TGF-β inhibitors: 2-(3-(6-Methyl-2-pyridinyl)-1H-pyrazol-4-yl)-1,5-naphthyridine (RepSox/RpS) and 4-(3-(2-pyridinyl)-1H-pyrazol-4-yl)-quinoline (LY364947) (Figure S6) on INS-1E and MIN6 cell proliferation (Figure 9a). First, we observed that treatment of INS-1E and MIN6 cells with TGF-β inhibitors alone did not affect cell proliferation [37] for the mouse islets. Application of harmine caused ~ a 2% labeling index as assessed using Ki67 labeling of insulin-positive cells. In contrast to the effects produced by the RepSox and LY364947 alone, both TGF-β receptor inhibitors induced a significant increase in the Ki67 labeling index in β-cells when combined with harmine or AC27. Proliferation rates expressed by the respective labeling indices obtained for AC27-treated cells averaged above the 12% range and were about 5% higher than those observed without TGF-β inhibition (Figure 9b,c). Furthermore, a combination of AC27 with TGF-β inhibitors markedly improved insulin/C-peptide secretion (Figure 9d,e) and reduced glucagon level after GSIS (Figure 9f). The results obtained are confirmed by confocal microscopy images (Figure S7). Importantly, this effect reached a maximal value between day 3 and 6 post-treatment and was stable up to 12 days after inhibitor action, similar to the other, well-known DYRK1A inhibitors (i.e., harmine, 5-IT) [14].

**Figure 10:**
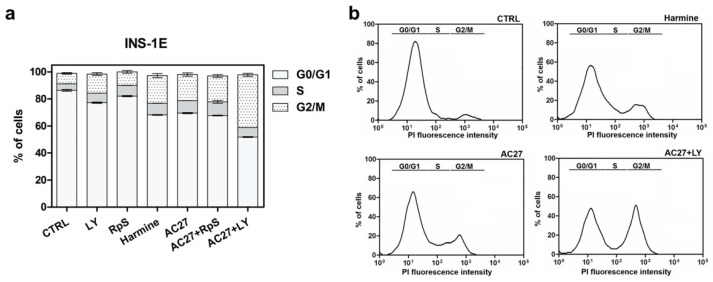
(**a**) Effects of the harmine, AC27 and AC27-TGFβ inhibitor combination on the cell cycle. (**b**) Histogram of INS-1E stained with propidium iodide showing DNA content distribution after the 24 h treatment with tested compounds (5 µM). G0/G1 and G2/M phase histogram peaks are separated by the S-phase distribution.

Physical, biological or chemical means can affect the DNA content and the cell cycle. There are three main phases in the cell cycle: the G0/G1 phase in which there is one set of paired chromosomes per cell, the S-phase in which DNA synthesis occurs and the amount of DNA is variable, and the G2/M phase characterized by two sets of paired chromosomes per cell, before cell division. The amount of DNA can be measured using dyes whose emission signal is proportional to the mass of DNA. In this case, propidium iodide was used. Cells were synchronized to a single-phase before the experiment. The cytometric analysis produced frequency histograms showing the phase of the cycle. The results are shown in Figure 10.

DYRK kinases play a key role in the control of cell proliferation and differentiation. DYRK1A and DYRK1B are negative regulators of the cell cycle that promote the transition to a cellular resting state. Furthermore, overexpression of human DYRK1A inhibits cell proliferation in many cell lines. After treatment with AC27 inhibitor alone and in combination with TGF-β inhibitors, an increase in the number of cells in the G2/M phase is observed. Compared to control cells, cells treated with AC27 in combination with LY364947 caused a 4-fold increase in the induction of cell division. Compared to harmine, this combination resulted in a significant increase in cells in the G2/M phase. The results obtained confirm that the tested compounds increase pancreatic β-cell proliferation. It has been shown to strongly stimulate proliferation in adult human pancreatic beta cells. Harmine and other DYRK1A inhibitors enable an increase in proliferation between 2–4%, and in combination therapies with TGFβ inhibitors and GLP-1 receptor agonists, this level increases to 5–8%. Despite the application of optimal conditions still, >90% of beta cells are not able to proliferate. This phenomenon has been attributed to various reasons, including terminal differentiation, quiescence and/or senescence, but these are still speculations and the mechanisms have not been explained [53].

### 3.7. DYRK1A Inhibitors Activity in the Advanced Pancreatic Islet Models (Hipsc, Isolated Islets) Hipsc

**Figure 11:**
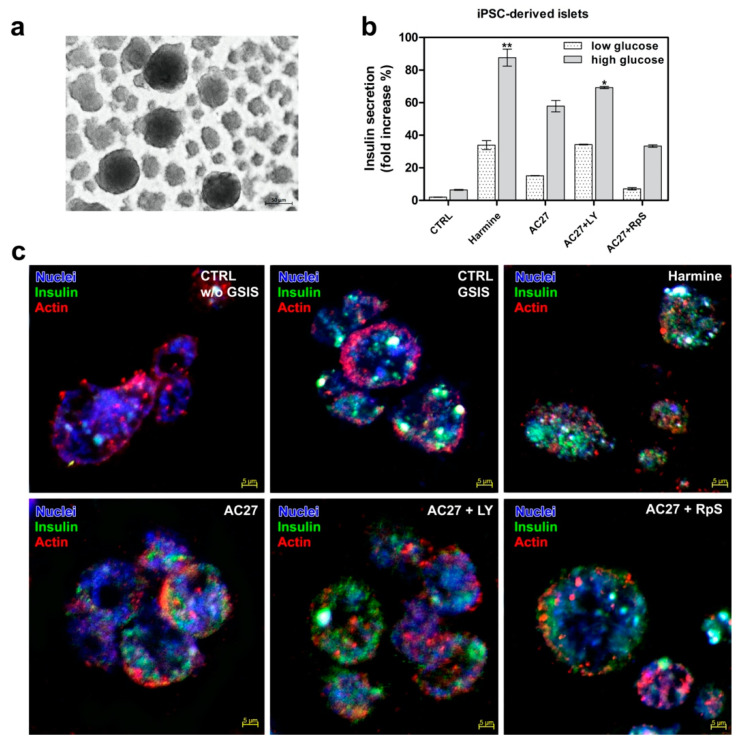
Development of hiPSC-derived β-cells organoids. (**a**) Morphology critical to directed differentiation-brightfield images of hiPSC spheres from seeding, day 21 of the differentiation protocol. Images show the morphology of the spheres important to ensure the correct directed differentiation to β-cells. (**b**) The insulin secretion assay was performed by incubating islets with harmine or AC27 (alone, or with RepSox or LY364947) at basal (2 mM, dotted bars) and high (25 mM, grey bars) glucose concentration. Values are normalized to the total amount of insulin, and insulin secretion is shown as a fold increase where the basal level is set at 1. Data are presented as the arithmetic mean ± SEM. (**c**) Representative immunostaining of hiPSC-like organoids stained for insulin (INS, green), actin (red) and Hoechst 33342 (blue). Scale bars, 10 μm. Data are expressed as mean ± SEM. * *p* < 0.05, ** *p* < 0.01, *** *p* < 0.001; as compared to control.

AC27 appeared most active from the investigated series; hence it was selected for the secondary studies in advanced islet models. Thus, to further validate the activity of AC27 in a more complex cellular assembly, we used the human induced pluripotent stem cells (hiPSC)-derived pancreatic organoids. We used a previously described hiPSC line generated from the peripheral blood mononuclear cells with non-integrating Sendai vectors for that purpose [54,55]. The hiPSC differentiation and islet organoid formation depends on transcriptional gene regulation involved in pancreas development and various growth factors and stem cell culture (Figure S8a). In this work, we differentiated hiPSC using the Pellegrini protocol with modifications [56]. The differentiation progress was determined in each step of this process, based on the expression of selected markers—FOX2A, SOX17, PDX-1 and pancreatic hormones (insulin, glucagon, C-peptide) (Figure S8b–e). Finally, we obtained the 40–100 μm sized, properly shaped organoid-like clusters on the Matrigel platform (Figure 11a). Of note, they contained functional insulin-expressing pancreatic cells and displayed a robust GSIS response (Figure 10g,h). Moreover, we observed a markedly increased insulin release in cells treated with harmine, AC27, AC27 + LY364947 and AC27 + RepSox combinations, respectively, comparing with the untreated control cells (Figure 11b, Figure S9).

This model was used to measure the effects of DYRK1A inhibition by the tested compounds. The possibility of obtaining a large number of mature, insulin-producing cells from hiPSC could provide an unlimited source of surrogate β-cells to replace damaged cells in diabetic patients of all types of diabetes. In this context, consistently with prior data on this model [56], we found that hiPSC-like organoids are functional insulin-expressing pancreatic cells (Figure 11b,c) that display a robust GSIS response over a range of physiologically relevant glucose concentrations and exhibit an elevated response to untreated pancreatic islets.

### 3.8. Isolated Mouse Islets as an Advanced Model for Preclinical Research

**Figure 12:**
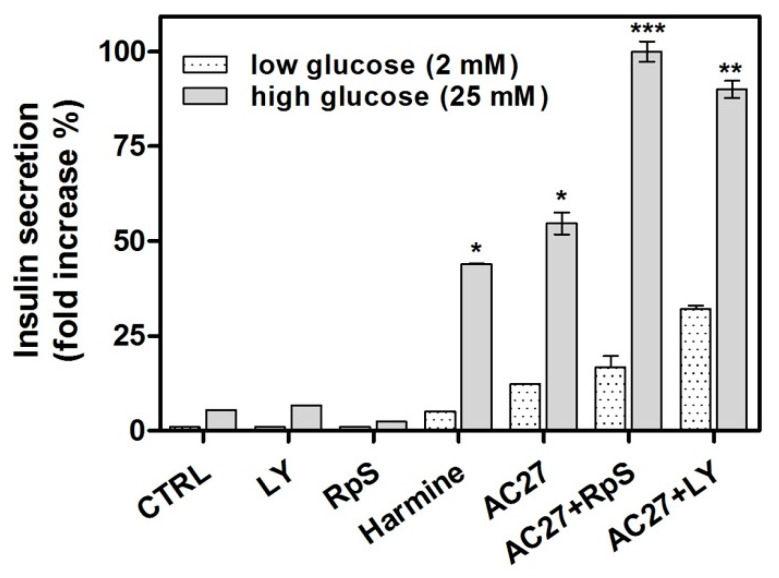
The activity of DYRK1A inhibitors in advanced models–isolated mouse islets. The insulin secretion assay was performed by incubating islets with the selected inhibitors (alone, with RepSox, with LY364947) at basal (2 mM, dotted bars) and high (25 mM, grey bars) glucose concentration. Values are normalized to the total amount of insulin, and insulin secretion is expressed as a fold increase where the basal level is set at 1. Data are presented as the arithmetic mean ± SEM. Data are expressed as mean ± SEM. * *p* < 0.05, ** *p* < 0.01, ***p < 0.001; as compared to control.

Post-isolation islet survival is a critical step for achieving successful and efficient islet transplantation. Following isolation, the round shape islet morphology was preserved (Figure S10). The double fluorescence cell viability stains assessed the viability of cultured islets. Freshly isolated islets have little or no central necrosis that would produce red fluorescence upon staining with propidium iodide. Most cells were viable according to the calcein-AM green fluorescence signal (Figure S10a). We also studied the distribution of the homeodomain proteins Pdx-1 and Nkx 6.1 in isolated mouse islets (Figure S10b,c). The presence of both Pdx-1 and Nkx 6.1. is detected only in the islets with mature insulin cell function. During early pancreatic development, cells that co-store insulin and glucagon are regularly detected. The vast majority of these did not reveal nuclear staining for either Pdx-1 or Nkx 6.1. On the other hand, it is known that the insulin-producing cells strongly display Pdx-1- and Nkx 6.1-positive nuclei [57,58]. Thus, our data suggest the appropriate characteristics and high functionality of isolated islets (Figure 12 and Figure S10d).

Next, we examined whether the cultured islets maintain their normal functions. We utilized the immunofluorescence and ELISA assays (Figure 12) to compare the functionality of the isolated mouse islet populations in situ and after isolation. We measured the insulin secretion into the culture medium from the islets of each group (control, LY364947 only, RepSox only, harmine, AC27 only, AC27 + LY364947 and AC27 + RepSox, respectively), under both conditions: low glucose and high glucose exposure (Figure 12). For glucose responsiveness, the GSIS was examined in all groups after 4 days in culture. Islets were stimulated by two consecutive exposures of low (2 mM) and high (20 mM) glucose concentrations (Figure 12). Exposure of mouse islets to a high glucose concentration on day 4 after treatment with DYRK1A inhibitors resulted in enhanced insulin secretion compared to the low-glucose groups (Figure 12). Importantly, the islets treated with AC27 demonstrated a 60%-fold increase in insulin secretion compared with control islets and ca. 15% higher secretion in case of application of harmine. When insulin secretion was normalized for total insulin content (Figure 12), the islets treated with the DYRK1A inhibitor AC27 released a higher percentage of total insulin, and the effect was potentiated for combinations of AC27 with TGF-β inhibitors LY364947 and RepSox (Figure 12).

**Figure 13:**
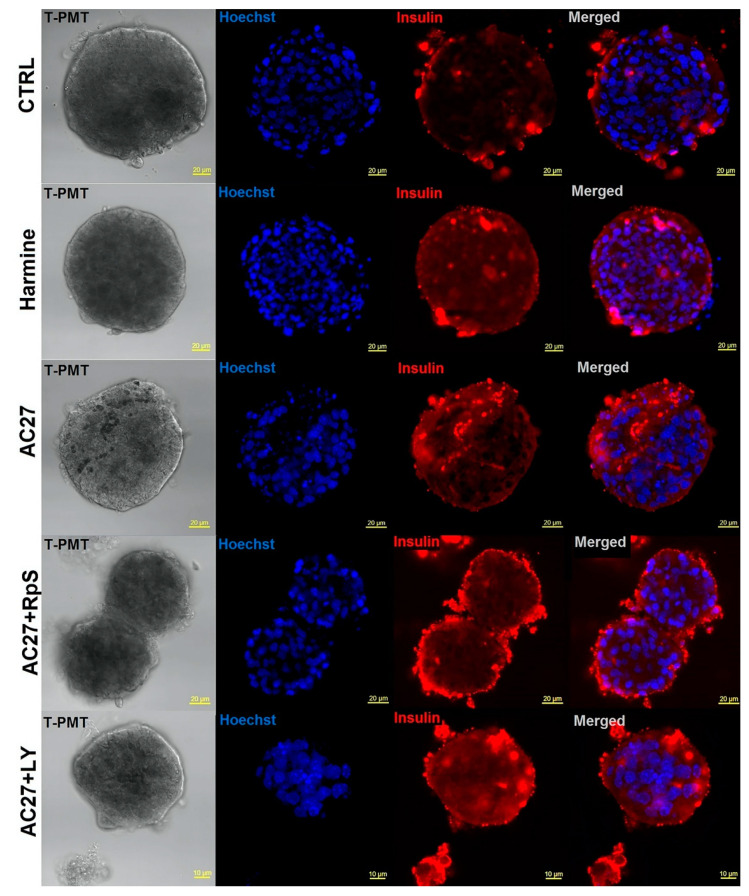
Representative insulin immunostaining of untreated islets after GSIS, and islets treated with harmine, AC27 alone, the AC27-LY364947 and the AC27-RepSox combination (red: insulin, blue: Hoechst 33342), scale bars, 5 μm and 10 μm.

To test the insulin content in situ, we stained the islets for insulin and imaged them by confocal microscopy. Once more, the insulin content was more outstanding in the AC27 treated islets compared to islets after GSIS only (Figure 13 and Figure S11). Furthermore, after treatment with AC27, the insulin secretion was significantly higher than observed for the untreated islets, which correlated with the higher insulin content/area in islets (in situ), a higher density of insulin secretory granules, and greater insulin content/volume in the isolated islets. Insulin (and free C-peptide) are packaged in the Golgi into secretory granules which accumulate in the cytoplasm. When the beta cell is appropriately stimulated, insulin is secreted from the cell by exocytosis. Thus, after GSIS the signal may be observed both inside islets, in periphery as well as outside the cells. Confocal images present mainly insulin accumulated inside islet and on periphery. The secreted amount of insulin was assessed by ELISA. Thus, using three independent approaches from both in situ and in vitro preparations, we obtained the evidence supporting the hypothesis of AC27 islets induced insulin secretion in the ex vivo model.

## 4. Discussion

In this work, the compounds that bind and inhibit DYRK1A [44] kinase have the ability to promote β-cell regeneration in advanced cellular models significantly. Increased β-cell mass and function by inducing proliferation of native or transplanted β-cells hold promise for a durable cure for diabetes. However, practical means are not yet available. It has been demonstrated earlier that DYRK1A inhibition has a positive effect on β-cell proliferation and insulin secretion. However, the best available small molecule inhibitor, harmine, is characterized by suboptimal kinase selectivity. We have thus selected a number of inhibitors of diverse scaffolds, characterized by better selectivity, and evaluated those for functional effect on β-cells.

AC27 induced expression of NFAT driven reporter, while earlier studies have demonstrated that conditional ablation of NFAT caused a reduction of β-cell mass in mouse models [59]. AC27 not only inhibited DYRK1A activity, but the inhibitor treatment resulted in a marked decrease of DYRK1A level in the cells. This was associated with increased proliferation of inhibitor-treated β-cells. Further, AC27 increased insulin and C-peptide secretion in response to glucose challenge without significantly affecting the glucagon level. The effects were significant, achieving more than 60% increase in insulin expression and long-term at the timescale of experiments. The effect reached a maximal level between day 3 and 6 post-treatment and was stable up to 12 days of treatment (persisting after inhibitor withdrawal). These results are consistent with the recent reports that DYRK1A inhibition induces human β-cell proliferation [14,34,35]. The effects of DYRK1A inhibition by AC27 were enhanced by simultaneous inhibition of TGF-β. Importantly, the results obtained in insulinoma cell lines were reproduced in more advanced pancreatic islet models. AC27 significantly improved glucose-induced insulin secretion in iPSC-derived β-cell organoids and isolated mouse pancreatic islets. The effect was also potentiated in combination with TGF-β inhibitors. Overall, our results strongly argue that DYRK1A inhibition with AC27 promotes β-cell proliferation and function, being superior to harmine, primarily in terms of target kinase selectivity (Table 2).

The induction of β-cell proliferation and function by small molecules promises future therapeutic utility. One could imagine the improvement of pancreatic function by inducing the proliferation of residual β-cells or pharmacological support of pancreatic islets upon transplantation. This offers an opportunity to regain β-cells destroyed by an autoimmune reaction leading to deficiency of endogenous insulin secretion (T1DM) and secondary abnormalities in the function of other pancreatic islet cells, such as abnormal glucagon release by α-cells. High glucagon levels after oral food intake are associated with postprandial hyperglycemia in patients with T1DM [60]. Moreover, an unbalanced glucagon and insulin secretion may contribute to glucose fluctuations and inadequate glucose control in type 2 diabetic patients [60]. Generating mature insulin-expressing cells with the same GSIS capability as endogenous β-cells and their survival following transplantation into ectopic sites in experimental host animals are future research challenges. Qader et al. reported that ghrelin (secreted from type A cells in the gastric mucosa) and nitric oxide (NO) have apparently parallel effects on insulin secretion (inhibitory) and glucagon secretion (stimulatory). These abnormalities in glucagon secretion may be due to lack of insulin, reduced insulin secretion by β-cells or a reduced suppressive effect of insulin on glucagon secretion [61]. Regenerative approaches, though still distant, could possibly result in a durable cure. Appropriate tool compounds, such as AC27 reported in this study, bring us steadily closer to regenerative therapy.

Future studies shall assess in vivo the ability of the reported compounds, alone and in combination, to regenerate the damaged pancreas and restore the blood glucose levels without negatively affecting other pathways. Another aspect of being investigated is the possible resistance to the β-cells growth-promoting agents in the hostile environment of the damaged pancreas and β-cells protection against autoimmune assault.

The structure of AC27 closely resembles mTOR inhibitor Torin2 (Figure 1). The latter compound significantly reduces β-cell viability already at 0.01 μM concentration while AC27 is non-toxic up to 5 μM concentration. Kinome profiling [44] demonstrates that AC27 inhibits mTOR and DYRK1A with similar strength. However, Torin2 is 40 times more potent towards mTOR compared to AC27 [62]. Significant activity against mTOR most likely underlines the reduction of β-cell viability upon Torin2 treatment. This information shall guide further optimization of AC27, which should be directed at further reduction of activity towards mTOR. Moreover, there is co-relation between DYRK1A and nitric oxide synthase expression [63], because DYRK1A inhibition might also results in a reduced NO generation which is a cytotoxic molecule for β-cells causing their dysfunctionality [64]. Thus, DYRK1A inhibition might result in an overall improvement of β-cell function by suppressing NO generation too the inhibition of NOS improves β-cell function in diabetic GK rats and human islets as well [65].

**Scheme 1:**
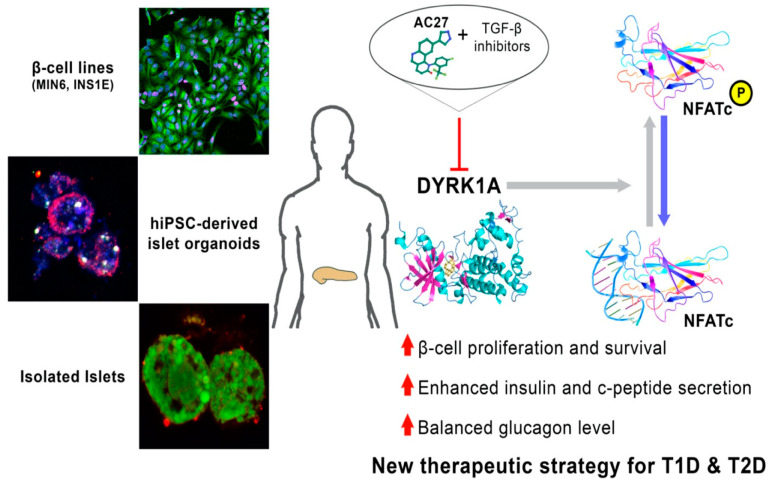
Summary of DYRK1A inhibition mediated by AC27 with molecular and cellular consequences.

In summary, we presented compounds that inhibit DYRK1A and thereby regulate insulin secretion and glucose-stimulated proliferation in cell cultures, organoid models and isolated pancreatic islets (Scheme 1). Such advancements suggest that small molecule-induced human β-cell proliferation is achievable in future clinical practice.

## Figures and Tables

**Table 1:** The dissociation constant (Kd) and inhibitory constant (Ki) values determined for the studied compounds and DYRK1A in MST and Cook assays, respectively.

Compound	MST	Activity Assay
	Kd [μM]	Ki [μM]
AC12	0.38 ± 0.06	0.10 ± 0.03
AC13	0.9 ± 0.2	0.15 ± 0.04
AC22	2.6 ± 0.4	1.6 ± 0.4
AC23	12.9 ± 2.6	1.2 ± 0.4
AC25	1.1 ± 0.6	1.0 ± 0.3
AC27	1.8 ± 0.5	0.2 ± 0.3
AC28	27 ± 17	3 ± 1
Torin2	0.3 ± 0.1	4.6 ± 1.8
Harmine	0.9 ± 0.3	0.11 ± 0.03

**Table 2:** The comparison of selected properties of harmine and AC27. Abbrev. In order: ↑/↑↑ = moderate/most potent.

		Harmine	AC27
Biochemistry	Ki	0.11 ± 0.03	0.17 ± 0.26
	Kd	0.9 ± 0.294	1.83 ± 0.543
	specificity [44]	no	yes
	off-targets [44]	yes	no
Cell lines	toxicity	no	no
	Ki67+	↑↑	↑
	Ki67+/insulin+	↑	↑
	insulin+	↑	↑↑
	glucagon+	↑↑	↑
	C-peptide+	↑	↑↑
hiPSC	insulin secretion (ELISA)	↑↑	↑
Isolated islets	insulin secretion (ELISA)	↑	↑↑

## Data Availability

Data are available upon reasonable request from the corresponding author. Additional data and results are provided in the Supplementary Materials.

## Supplementary Materials

The following are available online at https://www.mdpi.com/article/10.3390/cells10092263/s1, Figure S1. Structure of β-cell replication-promoting compounds; Table S1. Inhibitory constant (Ki) values determined for investigated compounds in the Cook assay. Data previously reported; Figure S2. Correlation between Kd and Ki values determined for DYRK1A-inhibitors; Figure S3. Relative cell viability of HEK293 cell line treated with AC compounds; Figure S4. Analysis of the influence of DYRK1A inhibitors on proliferation of INS-1E cells; Figure S5. The influence of DYRK1A inhibitors on INS-1E; Figure S6. Structures of TGF-β inhibitors. (a) RepSox. (b) LY364947; Figure S7. Representative confocal images of a Ki67 and insulin double-positive cells induced by the AC27-TGF-β inhibitor combination; Figure S8. Development of hiPSC-derived β-cells organoids; Figure S9. The insulin secretion assay; Figure S10. Isolated mouse islet; Figure S11. Representative insulin immunostaining of untreated islets; Figure S12. The influence of AC compounds on INS-1E and MIN6 functionality.
