# Supplementary material for: DYRK1A Kinase Inhibitors Promote β-Cell Survival and Insulin Homeostasis
Source: Cells. 2021 Aug 31;10(9):2263. doi: 10.3390/cells10092263 (PMC8467532; doi:10.3390/cells10092263)
Supplement: Supplementary file 1 [file cells-10-02263-s001.zip › cells-1351137 Supplementary for conversion.pdf]

Supplementary Materials for

DYRK1A kinase inhibitors promote β-cell survival and insulin homeostasis

Agata Barzowska[1], Barbara Pucelik[1], Katarzyna Pustelny[1], Alex Matsuda[1], Alicja Martyniak[2], Jacek Stepniewski[2], Anna Maksymiuk[3], Maciej Dawidowski[3], Ulli Rothweiler[4], Jozef Dulak[2], Grzegorz Dubin[1] and Anna Czarna[1*]

[1] *Malopolska Center of Biotechnology, Jagiellonian University, Gronostajowa 7A, 30-387 Krakow, Poland*

[2] *Department of Medical Biotechnology, Faculty of Biochemistry, Biophysics and Biotechnology, Jagiellonian University, Gronostajowa 7, 30-387 Krakow, Poland*

[3] *Department of Drug Technology and Pharmaceutical Biotechnology, Medical University of Warsaw, Banacha 1, 02-097 Warszawa, Poland*

[4] *The Norwegian Structural Biology Centre, Department of Chemistry, UiT, The Arctic University of Norway, N-9037 Tromsø, Norway*

*Correspondence: anna1.czarna@uj.edu.pl

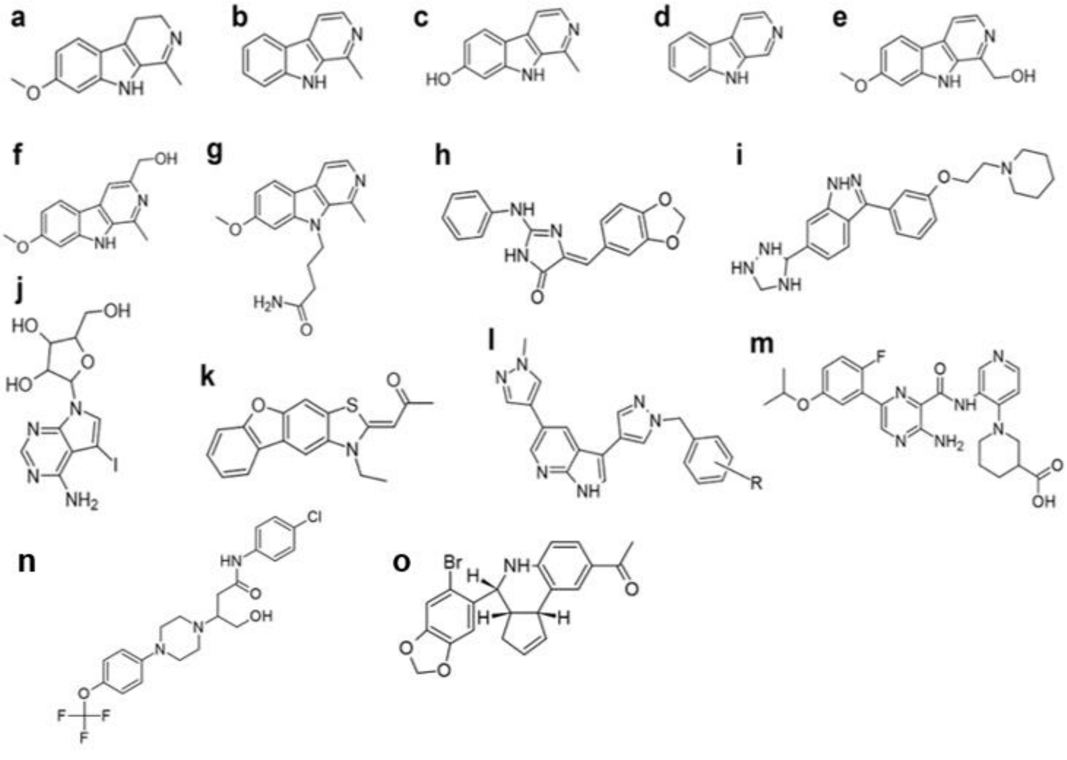

Figure S1. Structure of β-cell replication-promoting compounds. a. Harmaline, b. Harmane, c. Harmalol, d. Norharmane, e. Compound I, f. 4-(7-Methoxy-1-methyl-β-carbolin-9-yl)butanamide, g. L41, h. CC-401, i. 5-IT, j. INDY, k. BINDY, l. 7-azaindole derivatives, m GNF- 4877, n. VBIT-4, o G-1.

Table S1. Inhibitory constant (Ki) values determined for investigated compounds in the Cook assay. Data previously reported[1].

Compound	Activity assay
	**K_i [µM]***
AC12	0.104
AC13	0.168
AC22	>2.8
AC23	2.015
AC25	0.575
AC27	0.252
AC28	–
Torin2	–
Harmine	–

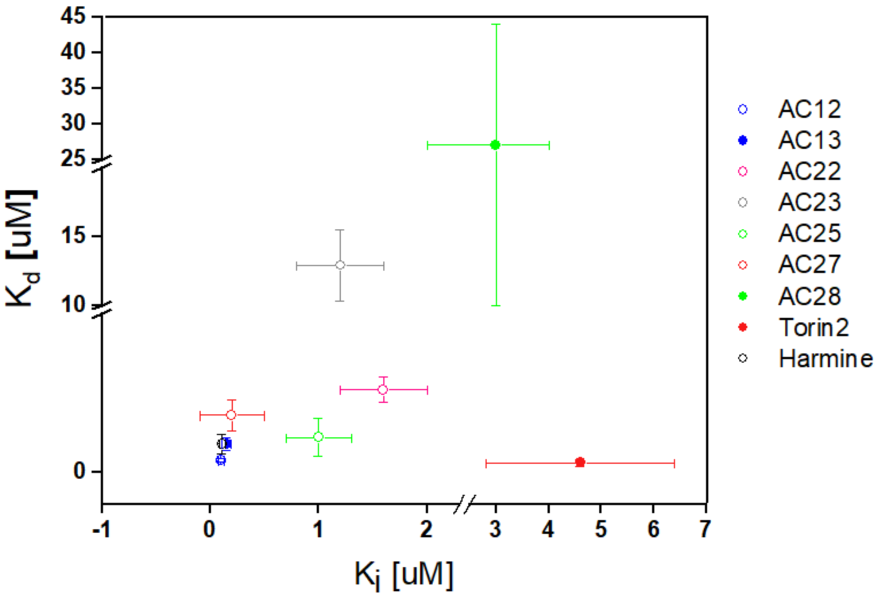

Figure S2. Correlation between Kd and Ki values determined for DYRK1A-inhibitors. The K_d values were determined by MST in a direct binding assay using constant concentration of fluorescent-labelled DYRK1A kinase domain (20 nM) titrated with increasing concentrations of compounds (15 nM-250 µM). The K_i were determined in Cook activity assay using fixed concentration of DYRK1A (0.5 µM), ATP (128 µM), synthetic peptide DYRKtide (RRRFRPASPLRGPPK, 1 mM) and increasing concentration of compound (20 nM - 200 µM). Data are expressed as mean±SD from 3 independent experiments.

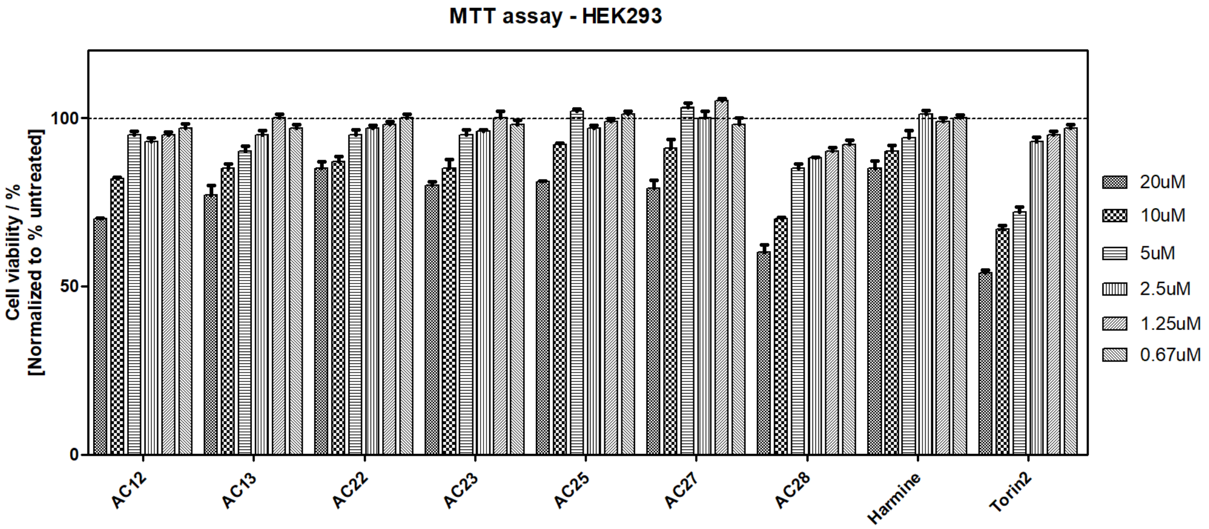

Figure S3. Relative cell viability of HEK293 cell line treated with AC compounds. MTT assay. The concentration of the compound ranged from 0.67 μM to 20 μM: AC12; AC13; AC22; AC23; AC25; AC27; AC28; Harmine**;** Torin2. The cytotoxicity of tested inhibitors was determined using an indirect method based on the determination of changes in normal cell function caused by the action of tested compounds. In the study, we used colorimetric assay MTT, which is related to the enzymatic activity of succinate dehydrogenase (mitochondrial enzyme). This enzyme, present in cells, converts the yellow soluble tetrazolium salt: 3-(4,5-dimethylthiazol-2-yl)-2,5-tetrazolium bromide to purple, insoluble crystals of the reduced formazan. The amount of formazan formed is proportional to the number of living cells because it is not formed in dead/metabolically damaged cells. The crystals are dissolved and then an absorbance measurement is performed.

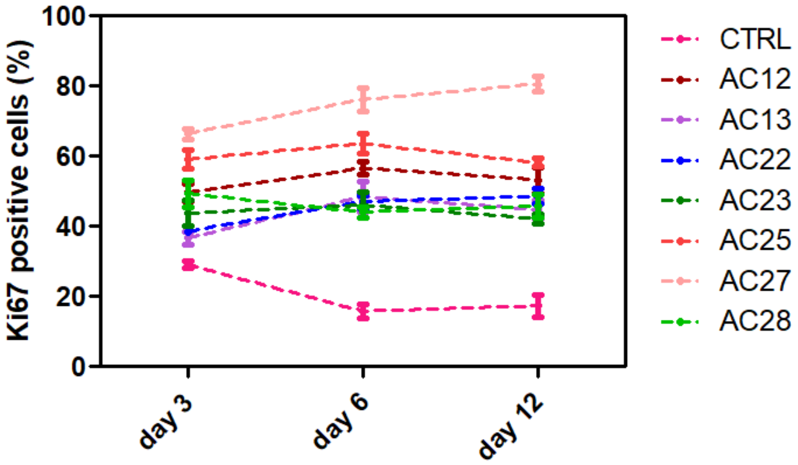

Figure S4. Analysis of the influence of DYRK1A inhibitors on proliferation of INS-1E cells. In Ki67 staining, 5 μM of each inhibitor used. Data are expressed as mean ±SEM; *P<0.05, **P<0.01, ***P<0.001 compared to the control.

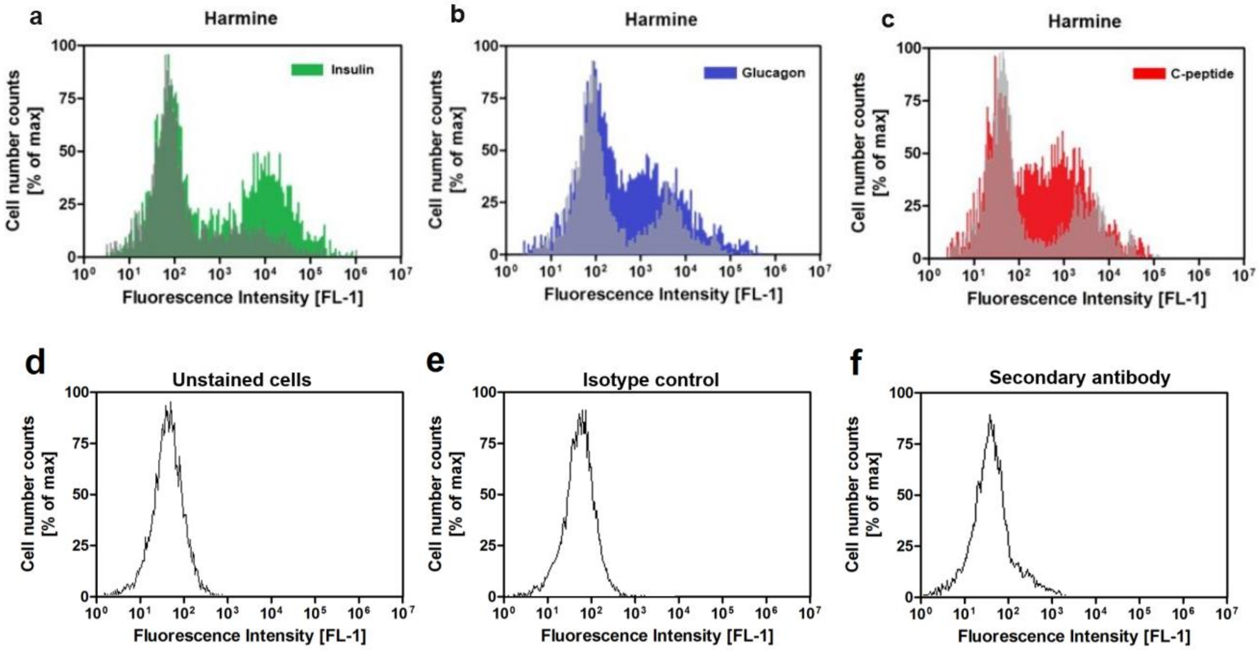

Figure S5. The influence of DYRK1A inhibitors on INS-1E. Flow cytometry analysis of insulin, C-peptide, and glucagon expression in INS-1E cell line. 24 h after incubation with 5 μM of the inhibitors and 72 h after applying the compounds, the cells were stained with the appropriate antibodies. a Insulin level in INS-1E cells treated with harmine (green) and control (black). b C-peptide level in INS-1E cells treated with harmine (red) and control (black). c Glucagone level in INS-1E cells treated with harmine (blue) and control (black). d unstained cells; e isotype control; f cells stained only with secondary antibody.

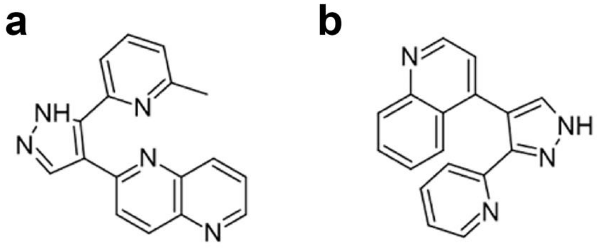

Figure S6. Structures of TGF-β inhibitors. a RepSox. b LY364947.

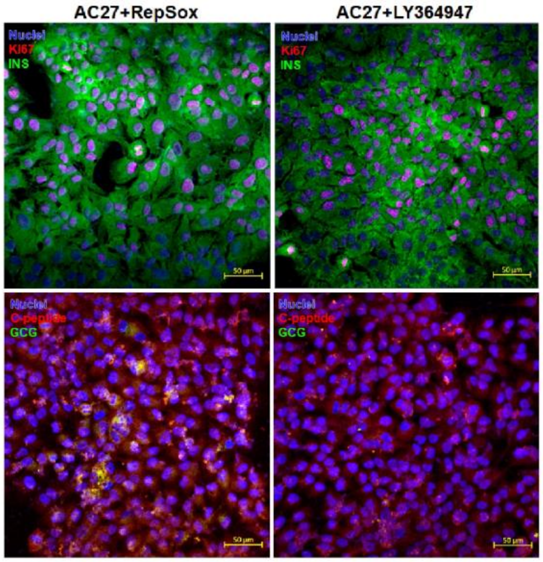

Figure S7. Representative confocal images of a Ki67 and insulin double-positive cells induced by the AC27-TGF-β inhibitor combination. The representative confocal images of C-peptide and glucagon double-positive cells induced by the AC27-TGF-β inhibitor combination in INS-1E cells. Scale bars, 50 μm.

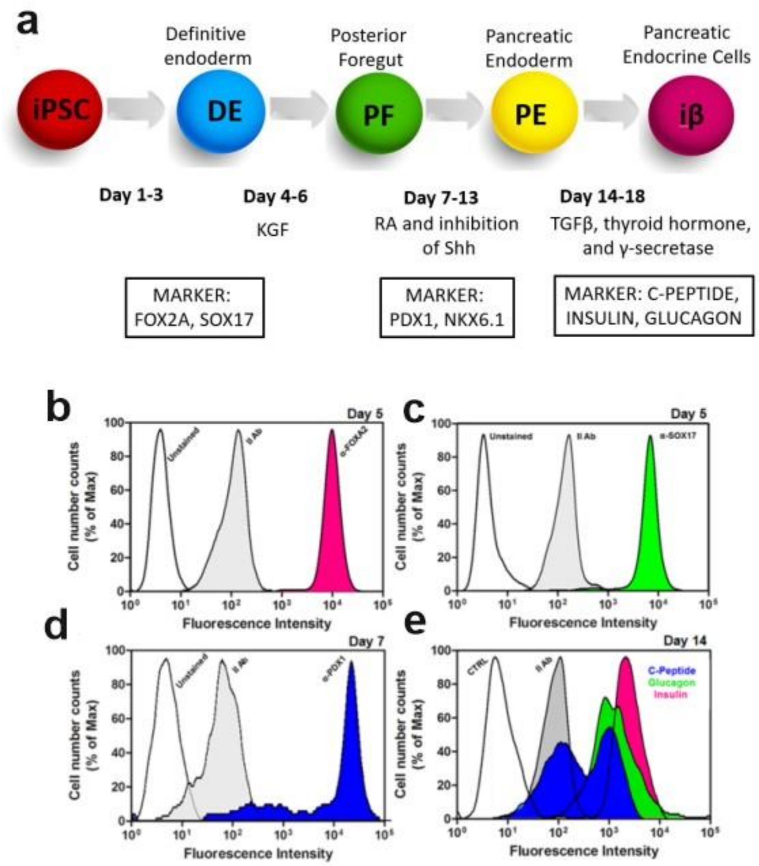

Figure S8. Development of hiPSC-derived β-cells organoids. a Schematic summary of differentiation protocol used to produce hiPSC-derived β-cell organoids. b-e Representative flow cytometry plot of differentiated iPSC cells stained for FOX2A b., SOX17 c., PDX1 d., and for C-peptide, glucagon, and insulin e.

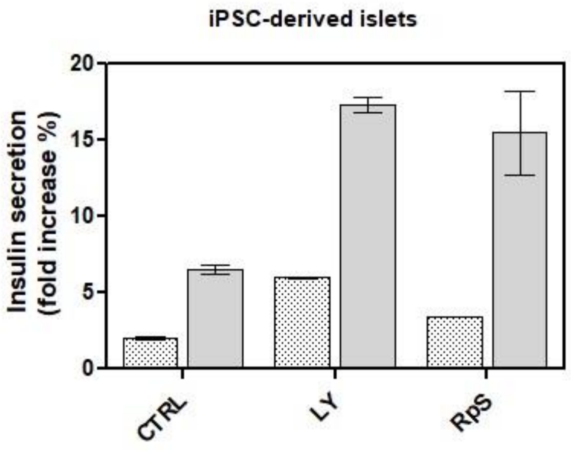

Figure S9. The insulin secretion assay. The insulin secretion assay was performed by incubation of iPSC-derived islets alone, or with RepSox or LY364947 at basal (2 mM, dotted bars) and high (25 mM, grey bars) glucose concentration. Values were normalized to the total amount of insulin and insulin secretion is expressed as a fold-increase where the basal level is set at 1. Data are presented as the arithmetic mean ± SEM.

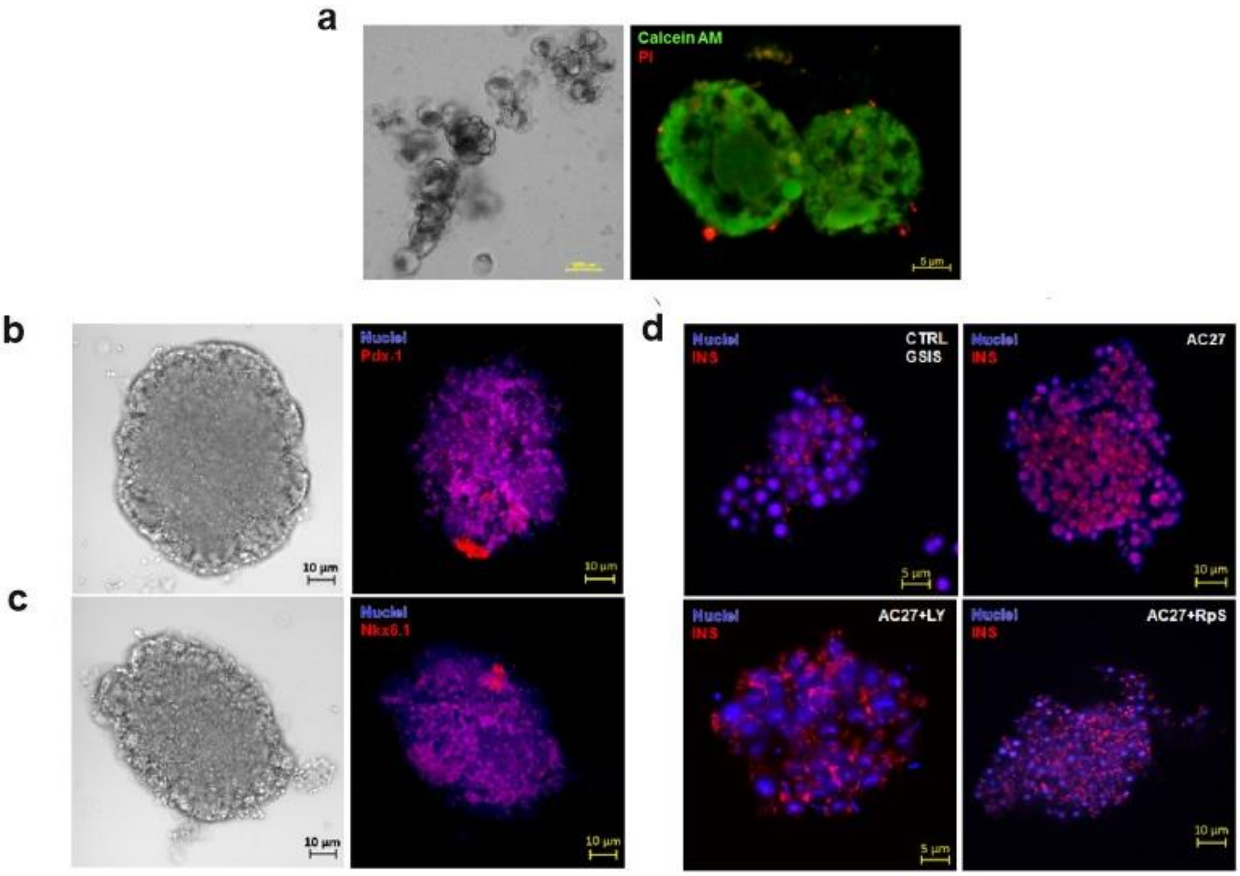

Figure S10. Isolated mouse islet architecture (left) and live/dead staining (right; green-Calcein AM; red-propidium iodide, PI), scale bars, 100 µm and 5 µm. b Immunofluorescence of Pdx-1 (red), scale bars, 10 µm. c Nkx6.1 homeobox protein isolated islets. Nuclei are stained in blue (Hoechst 33342), scale bars, 10 µm. d. Representative insulin immunostaining of untreated control islets after GSIS, and islets treated with AC27 alone, the AC27-LY364947 and the AC27-RepSox combination (red - insulin, blue - Hoechst).

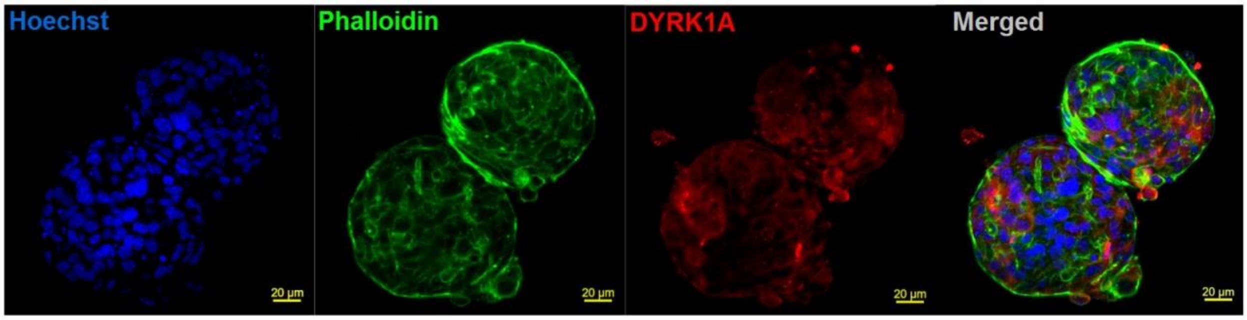

Figure S11. Representative insulin immunostaining of untreated islets. (red: DYRK1A, blue: Hoechst 33342, green: Phalloidin), scale bars, 5 µm and 10 µm.

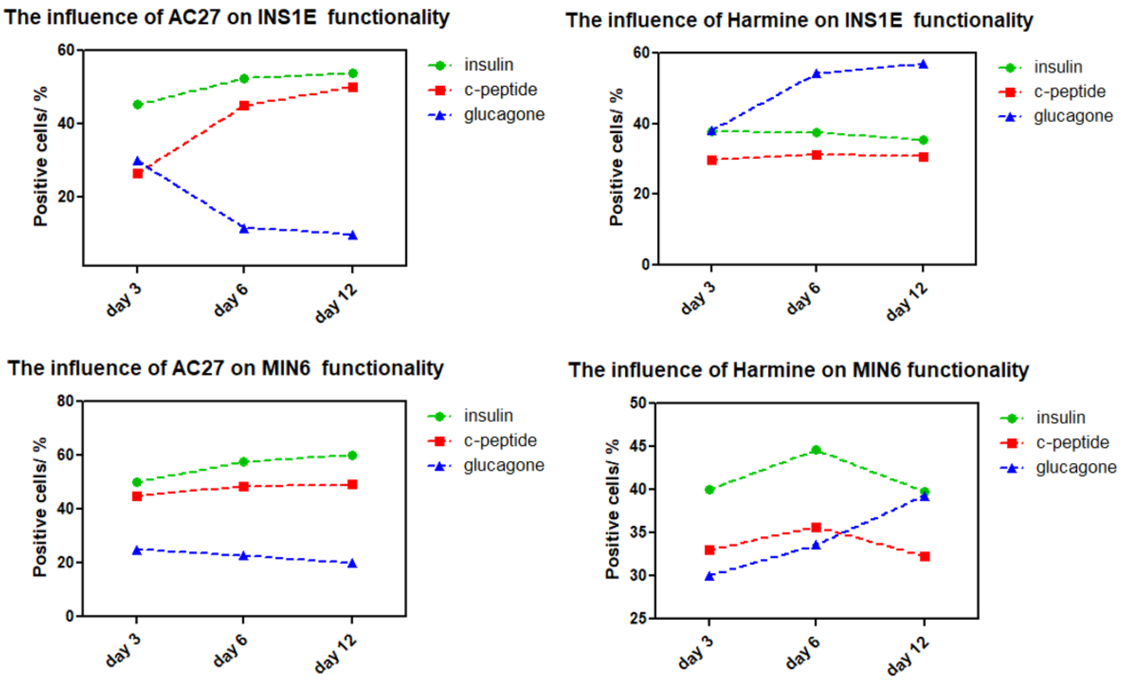

Figure S12. The influence of AC compounds on INS-1E and MIN6 functionality. Flow cytometry analysis of insulin, c-peptide and glucagon expression in MIN6 and INS-1E cell line. After incubating the cells with the inhibitors (5 µM, 24 h) and 3 days, 6 days and 12 days after adding the compounds, the cells were stained with the appropriate antibodies.

---

[1] Czarna, A. et al. Novel scaffolds for Dual specificity tyrosine-phosphorylation-regulated kinase (DYRK1A) inhibitors. Journal of Medicinal Chemistry 61, 7560-7572 (2018).
